# Knowledge, Attitude, and Self Care Practices Amongsts Patients WithType 2 Diabetes in Pakistan

**DOI:** 10.5539/gjhs.v8n7p1

**Published:** 2015-11-03

**Authors:** Muhammad Umer Ahmed, Haseeb Munaf Seriwala, Syed Hasan Danish, Ali Mahmood Khan, Marya Hussain, Misha Husain, Muhammad Mustafa Ahmed, Khurram Anis

**Affiliations:** 1 Ziauddin University and Hospital, Ziauddin Medical College, Karachi, Pakistan; 2 Dow University of Health Sciences (DUHS), Karachi, Pakistan; 3 Department of Community Medicine, Ziauddin University Karachi, Pakistan; 4 King Fahd Specialist Hospital Dammam (KFSHD), Saudi Arabia; 5 ZiauddinUniversity, Ziauddin Medical College, Karachi, Pakistan; 6 Milton Keynes General Hospital, UK

**Keywords:** type-2 diabetes, knowledge, attitude, selfcare practices, Pakistan

## Abstract

**Background:**

In this age, diabetes is one of the most prevalent, incurable diseases present. The aim of this study was to assess knowledge, attitude and self-care practicesrelated to diabetes in an urban population in Pakistan.

**Methods:**

A cross sectional survey to assess the knowledge and practices of people with diabetes was conducted in general urbanpopulace. People with diabetes were interviewed using a structured questionnaire from which data was collected. A total of 139 diabetics were included into the study. Basic knowledge about diabetes and its complications were assessed as well as the frequency of practices such as keeping a blood glucometer at home and checking blood sugar with it.

**Results:**

A total of n=139 participants fulfilling the inclusion criteria were recruited in the survey. Only 18.7% had knowledge regarding the complications of diabetes mellitus. Only 8.6% of participants checked their blood glucose levels at homeregularly, and only 4.3% visited their physiciansregularlyfor check-ups. With regard to practices, a minority attested to have changed their lifestyle and commit to basic practices in order to reduce diabetes related complications with women being more prone to changes than men.

**Conclusion:**

The results show that most participants had a negative attitude and very little knowledge regarding diabetes. There is a need for increased diabetes related education and for developing positive attitudes towards reduction of diabetes related complications. The Pakistani population is seen to be almost completely unprepared to fight against an increase in type 2 diabetes prevalence.

## 1. Introduction

In the current era of medicine, diabetes mellitus is one of the most prevalent, incurable and yet controllable diseases. In particular, type 2 diabetes mellitus has a number of life threateningcomplications which can be controlled with the proper control of blood sugars in these patients. The incidence of type 2 diabetes has been increasing in Pakistan but without any significant review into the various studies regarding the association between type 2 diabetes and the knowledge in these patients regarding their disease. Data suggests that patients with proper knowledge about diabetes, achieve better control over the disease and hence, the long term complications associated with it are avoided (McPherson, Smith, Powers, & Zuckerman, 2008). Adequate control of diabetes and achieving normal ranges of HbA1C are directly associated with better prognosis through the prevention of the long term sequelae of diabetes. *Education is likely to be effective if we know the characteristic of the patients in terms of knowledge, their attitude and practices about diabetes* (V. N. Shah, Kamdar, & N. Shah, 2009).

In 2011, the estimated prevalence of diabetes in Pakistan was approximately over 350 million and it is expected to be over 550 million by year 2030 (Whiting, Guariguata, Weil, & Shaw, 2011). *A study done in rural India showed that demographic transition due to improved living conditions in rural India was associated with a three-fold increase in the prevalence of diabetes. Increased upper body adiposity and physical inactivity showed significant association with this phenomenon* (Ramachandran et al., 2004). It has been proposed that by spreading knowledge among the patients regarding causative risk factors and the proper control of their blood sugar levels, people can effectively eliminate several risk factors that lead to severe diabetes such as obesity, and in turn reduce the chance of developing diabetes. Chronic hyperglycemia, represented by the levels of HbA1C, is directly associated to the long term renal, vascular and cardiac complications (Lind, Olsson, Rosengren, Svensson, Bounias, & Gudbjornsdottir, 2012; Skriver, Stovring, Kristensen, Charles, & Sandbaek, 2012). American Diabetes Association claims that an HbA1C level of less than 7% indicates good glycemic control.

*Another important factor leading to inadequate control of diabetes was highlighted by a study held in Rural Gujarat. The shocking fact was that physicians could spare very limited time for their patients and a search for complications was ignored by most. Foot care checking and self care motivation, the two main aspects of diabetes care were ignored by most of the treating practitioners*. (Shah, Kamdar, & Shah, 2009).

Self-Monitoring of Blood Glucose (SMBG) has been considered an important part of adequate blood glucose control since the early 1900’s (Bartlett, 1986). It is a method which makes the patient understand how the diet should be controlled and how much anti glycemic management is needed to optimize the control. Similarly, many patients know that ‘sweet’ foods are to be avoided, but knowledge regarding the specific sweet and rich in carbohydrate foods that they have to avoid is not clear among many patients. It is also a proven fact that life style modification is directly associated to better glycemic control (Mahdad, Boukortt, Benzian, & Bouchenak, 2014), but the prevalence of this information among diabetics has not been studied a lot in an urban area of Pakistan.

Hence, the aim of this study was to assess knowledge, attitude and self-care practicesrelated to diabetes in an urban population in Pakistan.

## 2. Methodology

A cross sectional survey to assess the knowledge and practices of people with diabetes were conducted in general population from June 2014 to October 2014. The actual sample size was 100 people with diabetes, which was calculated by using the standard formula for calculating sample size on the basis of prevalence:


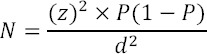


Prevalence was taken at 7%^R^. The bound of error was taken at 5% with 95% confidence interval. The sample size was inflated to 120 to exclude non-response and poorly filled questionnaires. Inclusion criteria involved patients with history of diabetes for more than 6 months. Non response and poorly filled questionnaires were excluded from the sample. The sample was selected from the general population through consecutive sampling technique, the questionnaire was based on previous studies conducted elsewhere and comprised of three portions of demography, knowledge of diabetes and attitudes and practices towards management of diabetes. The questionnaire was pilot tested to see if any changes needed to be made. After explaining the purpose of study and taking verbal consent, researchers conducted personal interviews. Data was entered on Microsoft excel and after editing was imported to SPSS version 20. Frequencies and percentages were taken out for categorical variables. Association between duration of diabetes, gender was taken out with knowledge and practices by application of χ^2^. P value less than 0.05 was taken as significant.

## 3. Results

A total of n=139 participants fulfilling the inclusion criteria were recruited in the survey. Males comprised 43% (n=60) and females were 57% (n=79) with the mean age of the sample being43 +/- 16 years. Sixty seven percent participants (n=91) were married, and the number of children per participants averaged 3.3 (2.6). Out of the total sample 10% (n=12) participants suffered from co-morbid diseases. When duration of diabetes was assessed majority participants 53% (n=73) had a history of 5 years and below, 29% (n=41) were in the category of 5 to 15 years and remaining 18% (n=25) had it for more than 20 years. Fifty seven percent, n=79 participants were taking insulin for managing diabetes. Family history of diabetes was present in 43.2% (n=60) participants. Only 9.4% (n=13) were ever admitted in hospital because of diabetes.

### 3.1 Knowledge Regarding Diabetes Mellitus

When enquired of whether diabetes result from high blood glucose, 28.8% (n=40) said yes, and another 20.1% (n=28) said it is because of failure of body to produce insulin. Likewise, when asked whether diabetes is a preventable disease or not, 12.9% (n=18) replied that it is preventable. Only 18.7% (n=26) had knowledge regarding the complications of diabetes mellitus.

### 3.2 Attitudes and Practices Regards to Management of Diabetes Mellitus

When participants were inquired about the presence of a glucometer at home for monitoring of blood glucose only 23% (n=32) replied in the affirmative and out of them only 8.6% (n=12) regularly checked their blood glucose at home. Only 4.3% (n=6) regularly visited their physicians at 3 months interval and another 72.6% (n=101) rarely visits their physician for follow up. For visit to Eye specialist only 10% (n=14) visited them every year and 83% (n=114) had never visited an eye specialist.

Out of the total participants, 20% (n=28) worked upon maintaining an ideal weight. Regular physical exercise was done by 8.6% (n=12) individuals. Only 2.9% (n=4) had modified their life style in order to control diabetes. This was evident as only 4.3% (n= 6) were following a proper diet plan and merely 7.9% (n=11) were taking their medicines on time. Proper cutting of nails was done by only 5.8% (n=8) participants. Conflicting observation was seen when control of diabetes was enquired from the participants, 46% (n=64) said it is always under control while only 4.3% (n=6) said it is never under control. When the impact of diabetes on the participants life was assessed, 67% (n=93) said it has not affected while 10% (n=14) said that diabetes has a profound effect on their daily life.

**Table 1:** Association of Gender with attitudes and practices related to management of Diabetes Mellitus

		Gender				P value

		Males (n= 60)		Females (n=79)		

		n	%	n	%	
**Glucometer at Home**	Yes	7	21.9	25	78.1	**0.003**

	No	51	51.5	48	48.5	

**Regular Blood sugar monitoring**	Yes	4	33.3	8	66.7	0.309

	No	56	45.5	97	54.5	

**Regular Physician visits**	Every 3 months	1	16.7	5	83.3	**0.008**

	Every 6 months	8	80	2	20	

	Every 12 months	11	52.3	10	47.7	

	Rarely	40	44.4	50	55.6	

**Medication on time**	Yes	2	18.2	9	81.8	**0.005**

	No	58	46.4	67	53.6	

**Life style Modifications**	Yes	1	25	3	75	0.403

	No	59	44.7	73	55.3	

**Proper Diet Plan**	Yes	0	0	6	100	**0.028**

	No	60	46.2	70	53.8	

**Regular Physical activity**	Yes	4	33.3	8	66.7	0.318

	No	56	45.2	68	54.8	

**Maintaining Ideal Body Weight**	Yes	11	39.3	17	60.7	0.360

	No	49	45.4	59	54.6	

**Proper nail cutting**	Yes	3	37.5	5	62.5	0.497

	No	57	44.5	71	55.5	

**Figure 1:**
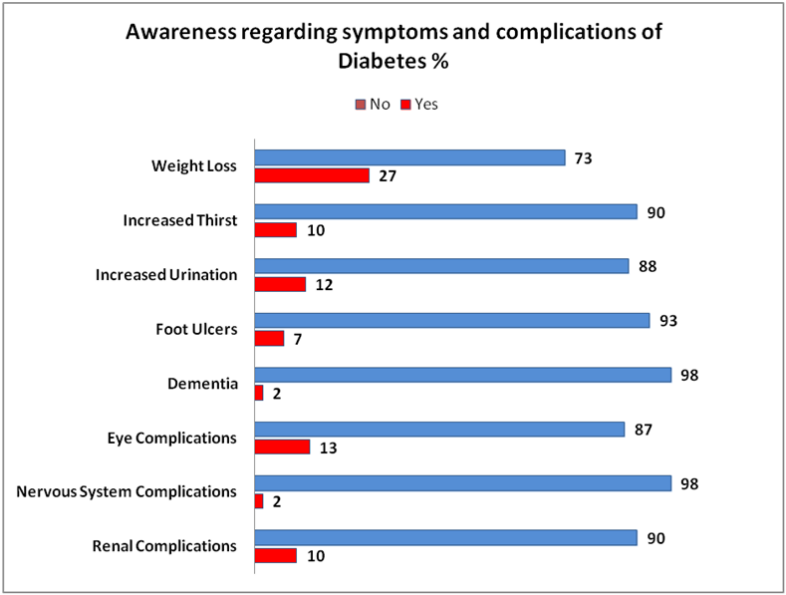
Knowledge regarding symptoms and complications of diabetes mellitus

**Table 2:** Association of duration of diabetes with attitudes and practices related to management of Diabetes Mellitus

	Duration of Diabetes						P value

	Less than 5 Years (n=73)		5–15 Years (n=41)		Above 15 Years (n=25)		

	n	%	n	%	n	%	
Glucometer at Home	Yes	21	65.6	1	3.2	10	31.2	0.001

	No	52	52.5	36	36.4	11	11.1	

Regular Blood sugar monitoring	Yes	12	100	0	0	0	0	0.004

	No	61	48	41	32.3	25	19.7	

Regular Physician visits	Every 3 months	3	43	2	28.5	2	28.5	0.322

	Every 6 months	8	66.7	4	33.3	0	0	

	Every 12 months	4	40	3	30	3	30	

	Rarely	42	42	32	32	20	20	

Medication on time	Yes	8	57	3	21.5	3	21.5	0.729

	No	65	51.5	38	30.1	22	18.4	

Life style Modifications	Yes	4	57	3	43	0	0	0.131

	No	69	52.3	38	28.8	25	18.9	

Proper Diet Plan	Yes	7	78	2	22	0	0	0.483

	No	66	50.8	39	30	25	19.2	

Regular Physical activity	Yes	6	40	5	33	4	27	0.137

	No	67	54	36	29	21	17	

Maintaining Ideal Body Weight	Yes	26	84	5	16	0	0	0.001

	No	47	43.5	36	33.3	25	23.1	

Proper nail cutting	Yes	8	66.7	3	33.3	0	0	0.384

	No	65	50.8	38	29.7	25	19.5	

## 4. Discussion

According to a study by Shaw et al., as of 2010, the number of people with diabetes in Pakistan was over 7 million, with these numbers expecting to rise at an annual rate 334,000 which is the fourth highest rate of increase, behind only China, India and the U.S.A. By 2030, it is estimated that the number of people with diabetes will reach an astonishing number of near 13.8 million. It can be seen that the number is projected to almost double in the coming years (Shaw, Sicree, & Zimmet, 2010). One in twenty adult deaths in developing countries is diabetes-related (Roglic et al., 2005). Hence, it is of utmost importance that the knowledge and awareness of diabetes in Pakistani diabetic patients be analyzed. Increased knowledge and awareness of diabetic patients about diabetes has been positively correlated with better glycemic control and a reduction in diabetes related complications (Panja, Starr, & Colleran, 2005). Thus, this is an area of great importance in the attempt to curb the adverse effects that diabetes causes in an individual’s life.

Several studies have concluded that there is poor knowledge about diabetes in the normal populace (Islam et al., 2014) and even for people with diabetes (Shah, Kamdar, & Shah, 2009; Al-Maskari, El-Sadig, Al-Kaabi, Afandi, Nagelkerke, & Yeatts, 2013; Ardena, Paz-Pacheco, Jimeno, Lantion-Ang, Paterno, & Juban, 2010; Hasan, Zia, & Maracy, 2004; Rafique, Azam, & White, 2006). One study indicated that educational level of respondents was associated with knowledge. A likely explanation is that those of a higher academic level (and hence of higher socioeconomic status) have a greater chance of obtaining knowledge from the mass media, books and the internet. In addition, they have fewer barriers in communicating with the health care team, and may have a good grasp of information. Expectedly, patients with no formal education were the least knowledgeable in this research (Jackson, Adibe, Okonta, & Ukwe, 2014). Even in studies done regarding family physician’s knowledge ofdiabetes was regarded as low (Shera, Jawad, & Basit, 2002; Bin Afif & Al-Mubarak, 2006). The results obtained concur with the results of these previous studies. Patients showed a low level of knowledge when 28.8% stated that high blood glucose leads to diabetes, and only 20.1% said that diabetes is due to the body’s failure to produce insulin. In addition, only a mere 12.9% knew that diabetes is preventable. In a study in the UAE by Al-Maskari et al. (Al-Maskari, El-Sadig, Al-Kaabi, Afandi, Nagelkerke, & Yeatts, 2013), roughly one third knew that diabetes is preventable. This shows a major difference between basic knowledge about the disease in a developed country such as UAE and a developing country like Pakistan. Few people had any idea regarding the complications of diabetes. The most well-known adverse effect known was weight loss, which was known to 27% of patients, followed by eye complications, known by only 13% of patients. Virtually none knew about dementia and nervous system complications. In some cases, patients were reported to know more of the consequences of diabetes (Shah et al., 2009; Panja et al., 2005; Islam et al., 2014; Al-Maskari et al., 2013; Ardena et al., 2010; Hasan et al., 2004; Rafique et al., 2006; Chasuk, Brantley, & Martin, 2001), similar findings were reportedin studies conducted elsewhere. The most notable of these was by Rafique et al. which showed a severe lack of diabetes knowledge within Pakistan (Rafique et al., 2006). Co-relation between knowledge and glycemic control was difficult to assess as most patients had very little knowledge. Other studies showed conflicting results relating to knowledge and glycemic control (Panja et al., 2005; Ezenwaka & Offiah, 2003).

As well as having knowledge of diabetes, self-management of diabetes is also vital for patients (Lombardo Salzano, Messina, & De Luca, 2003). The attitude and practices of each patient in order to maintain their disease has been subject to research in several different settings (Islam et al., 2014; Al-Maskari et al., 2013; Ardena et al., 2010; Rafique et al., 2006; Azar, Malha Zantout, Naja, Younes, & Sawaya, 2013; Ku & Kegels, 2014; Zhong, Tanasugarn, Fisher, Krudsood, & Nityasuddhi, 2011). Maintaining ideal weight was the most worked upon in practices to manage diabetes. This coincides with weight loss being the most well-known complication. Oddly, only 8.6% of individuals regularly monitored their blood glucose levels. This is in direct contrast to other studies where a much larger proportion of patients regularly monitored their blood glucose (Rafique et al., 2006; Ku & Kegels, 2014; Zhong et al., 2011), and this shows a significant difference in mindset of the selected population to those of previous studies. Few patients even kept glucometers at home. Compliance in the form of regular physician visits and taking medication on time was quite low. Several studies correlate this lack of compliance to decreased knowledge, education and a negative attitude towards their disease (Islam et al., 2014; Ku & Kegels, 2014; Zhong et al., 2011). These alarming and problematic findings may have severe detrimental effects in Pakistan, as it is expected for the people with diabetes in Pakistan to escalate to the fourth highest in the world (Shaw, Sicree, & Zimmet, 2010). Complications and diabetes related deaths may become commonplace.

Where it has been found that the level of knowledge is substantially low, it is also important to find the factors that have led to this. On analysis, several statistically significant differences have been found in practices, based on gender as well as duration of having the disease. Significant difference between males and females based on certain basic practices such as keeping a glucometer at home were observed. In addition, there was also difference for regular physician visits, taking medications on time and having a proper diet plan. This may be due to the fact that diabetes is relatively asymptomatic; hence, people find that there is no need for any type of change in life-style, rather, they believe that they are perfectly healthy (Brown, Harris, Webster-Bogaert, Wetmore, Faulds, & Stewart, 2002).

Statistically significant differences were also found for those having diabetes mellitus for different durations. Overall, it was noted that those diabetics that had diabetes for less than five years took more care and were more involved in practicing basic diabetic actions to prevent further consequences. Significant difference was found in keeping a glucometer at home, monitoring blood glucose levels and maintaining ideal weight. This could be because after time people feel that they are less affected by diabetes and thus create indifference towards their diabetes (Brown et al., 2002). Folklore is also a large problem in communities and hence leads to people committing to practices which may or may not help them (Rafique et al., 2006).

As patients were only taken from the urban population of Karachi, it is difficult to use the findings as a base for the entire country of Pakistan. The smaller sample size was a large limitation, in that a complete analysis of the entire population couldn’t be evaluated.

Our study shows a severe lack of concern of people with diabetes to their disease. With increasing numbers of diabetics, our study shows the need for change in attitude of people with diabetes in Pakistan. Therefore, the disease of diabetes shouldn’t be taken lightly, as it hasbeentakenfor many years in the country. Health officials need to make a solid plan to educate all people in Pakistan, not just those with diabetes. In order to create a more positive attitude towards diabetes, general practitioners need to explain to each person with diabetes how their lives are affected by diabetes and the potential consequences. Hospitals must teach patients what best practices they can do to prevent these potential consequences. With these basic changes, the general view of diabetes may change very quickly thus, diabetes can be controlled in the Pakistani population.

## 5. Conclusion

An eye-opening lack of knowledge, a negative attitude and a grave indifference toward diabetic practices is the current diabetic situation in Pakistan. Hence, educating the populace about diabetes should become one of the major goals for health practitioners. As there are many problems in educating in urban and rural areas (Thepwongsa, Kirby, Paul, & Piterman, 2014), the health sector in Pakistan faces a major task. With educationand awareness, a positive attitude may develop. People will then pay more concern to diabetic practices. This way, the Pakistani populace, which is expected to become the fourth highest in the world, can become better equipped to battle with diabetes and its complications. At the current status-quo however, Pakistan is unprepared for the large diabetes prevalence it is expected to have.
